# Citizen science in monitoring food environments: a qualitative collective case study of stakeholders’ experiences during the Local Environment Action on Food project in Alberta, Canada

**DOI:** 10.1186/s12889-022-13030-1

**Published:** 2022-04-06

**Authors:** Breanne L. Aylward, Krista M. Milford, Kate E. Storey, Candace I. J. Nykiforuk, Kim D. Raine

**Affiliations:** 1 School of Public Health, University of Alberta, 4-308 Edmonton Clinic Health Academy, 11405-87 Avenue, Edmonton, AB T6G 1C9 Canada; 2 School of Public Health, University of Alberta, 8303-112 Street, Edmonton, AB T6G 2T4 Canada

**Keywords:** Citizen science, Health promotion, Community participation, Child health, Food, Nutrition policy

## Abstract

**Background:**

Citizen science bears potential to build a comprehensive view of global food environments and create a broader discussion about how to improve them. Despite its potential, citizen science has not been fully utilised in food environment research. Thus, we sought to explore stakeholders’ experiences of the Local Environment Action on Food (LEAF) project, a community-based intervention that employs a citizen science approach to monitoring food environments.

**Methods:**

We used a qualitative collective case study design to explore citizen science through the LEAF process in seven communities in Alberta, Canada. Data generating strategies included semi-structured interviews with citizen scientists (*n* = 26), document review of communities’ Mini Nutrition Report Cards (*n* = 7), and researcher observation. Data were analyzed in a multi-phase process, using Charmaz’s constant comparison analysis strategy.

**Results:**

Analysis revealed two main themes: relationship building and process factors. Communities used three interconnected strategies, engaging the right people, treading lightly, and reaching a consensus, to navigate the vital but challenging relationship building process. Process factors, which were influences on the LEAF process and relationship building, included the local context, flexibility in the LEAF process, and turnover among LEAF community groups.

**Conclusion:**

Citizen science through the LEAF project supported the creation and application of food environment evidence: it enabled residents to collect and interpret local food environment data, develop realistic recommendations for change, and provided them with an evidence-based advocacy tool to support the implementation of these recommendations. We recommend a web application that enables independent community food environment assessments. Such a tool could stimulate and sustain citizen involvement in food environment efforts, helping to build the necessary evidence base and promote the creation of healthy food environments.

## Background

Poor diet is a leading behavioural risk factor for death worldwide, including in Canada [1]. Childhood is viewed as a critical intervention period for improving diet quality because eating practices developed at a young age often track into adulthood [2, 3]. Children and youth’s eating practices are shaped by numerous factors, including food environments; that is, the collective and contextual conditions in which food choices are made [4]. Although associations between children and youth’s food environments and diet-related outcomes have been demonstrated [5–7], more research is needed to develop a comprehensive understanding of the status and impact of children and youth’s food environments.

One strategy to build the food environment evidence base is citizen science, an approach that engages residents in research activities [8]. Although similar to community-based participatory research (CBPR), citizen science provides flexibility to engage individuals in less intensive research roles than offered by CBPR [9]. Organizations and researchers have created principles and best practices to provide guidance for all citizen science projects [10, 11], as well as various typologies to classify the broad range of activities within citizen science. For example, King et al. [12] outline three approaches within citizen science: 1) “for the people” projects, where residents donate specimens for biomedical research; 2) “with the people” projects, where residents assist in collecting data; and 3) “by the people” projects, where residents are more fully engaged as research partners and change agents. Beyond providing a time and resource efficient method to obtain substantial amounts of data, citizen science has the potential to enhance population and public health research and practice [9, 13]. By including lay perspectives and experiences, citizen science could produce more robust knowledge and promote locally relevant, effective health interventions (see A.C. King et al. [12] for a comprehensive list of benefits). Importantly, citizen science approaches can provide numerous benefits to non-researchers, such as empowering residents and building community capacity to create local action [9].

Despite broad potential, citizen science has not been fully utilised in food environment research and practice [14, 15]. A comprehensive overview of the current status of food environments may spur a broader discussion about how to improve the healthfulness of food environments and promote community-level action. Thus, this study aimed to explore participants’ experiences using a citizen science approach to monitoring and acting on children and youth’s food environments through the Local Environment Action on Food (LEAF) project. Impacts of the LEAF project are reported elsewhere [16].

### Setting: the Local Environment Action on Food (LEAF) project

The Local Environment Action on Food (LEAF) project is a community-based intervention implemented in 17 communities across Alberta, Canada. The term “community” can be defined in several ways, such as by a single neighbourhood or by non-geographical communities (e.g., people with a shared passion or goal) [17]. Although each “community” in this study was a municipality of varying size (see Table 1), we refer to LEAF as a community-based intervention because it could be implemented to align with other definitions of community. Part of a broader provincial strategy, which includes the *Alberta Nutrition Report Card on Food Environments for Children and Youth* (provincial NRC) [18], LEAF employs a “by the people” citizen science approach [12] to engage residents in monitoring and acting on local food environments and nutrition policies relevant to children and youth. Following Brennan et al. [19], LEAF evaluates five food environments: the physical, communication, economic, social, and political environments. Broadly, these environments correlate to food availability, messaging, affordability, norms, and rules, respectively. LEAF is a voluntary, collaborative effort between four main players: the research team (the principal investigator, the research project coordinator, and multiple research staff and students), the organizational partners (the province-wide health delivery system), the community partners (communities that chose to participate in LEAF), and the individual partners (staff members from the organizational partner and other involved community members). Staff members from the organizational partner (referred to as LEAF project leads) typically introduced LEAF to existing community groups that had displayed an interest in healthy eating. Interested communities worked with the LEAF research team in an iterative and recursive process (described below) to co-create a *Mini Nutrition Report Card on Food Environments for Children and Youth* (Mini NRC). The Mini NRC aimed to assess the status of each community’s food environment and act as a tool to advocate for locally informed recommendations for change.

**Table 1:** Community profiles

	Community A	Community B	Community C	Community D	Community E	Community F	Community G
Approximate Population _2016_^a^	950	950	4000	15,000	65,000	6000	2500
Population density per square km ^a^	352.6	259.9	315.8	777.3	564.6	405.4	197.9
Location^b^	Central Alberta	Central Alberta	Central Alberta	Southern Alberta	Southern Alberta	Central Alberta	Northern Alberta
Approximate distance to nearest medium/large urban population centre (km)^c^	140	180	200	100	0	100	20
Median age of population (years) ^a^	54.3	44.0	38.2	35.0	40.6	44.3	30.0
Unemployment rate (%)^a^	8.0	10.4	8.1	9.2	10.0	9.1	10.3
Median income persons over 15 ($)^a^	34,080	35,968	40,855	41,358	36,819	36,244	45,978
Education: no certificate, diploma or degree (%)^a^	18.6	32	19.4	27.4	21.0	23.9	19.6
Education: postsecondary certificate, diploma, or degree (%)^a^	48.3	42.7	52.0	38.2	47.6	47.0	31.0
Total visible minority population (%)^a^	1.2	6.8	6.6	36.8	6.8	5.7	2.1
Approximate time to complete the LEAF process (months)	8	8	14	14	14	18	20
Sector of employment: LEAF Project Lead(s)	Public Health Dietitian	Public Health Dietitian	Public Health Dietitian	Public Health Dietitian and Health Promotion Facilitator	Public Health Dietitian	Public Health Dietitian	Public Health Dietitian, Health Promotion Facilitator, and Wellness Coordinator

^a^[20]

^b^[21]

^c^[22]

### The LEAF Process

LEAF engagement followed an eight-step process: orientation, choosing areas of interest, data collection, data analysis, validation meeting, recommendation meetings (with and without the research team), and a community launch. These steps align with King et al.’s [12] “by the people” citizen science approach, where residents are research partners that contribute to data collection and analysis, as well as knowledge mobilization.

#### Step 1: orientation

The research project coordinator hosted in-person orientation workshops in each community, providing details on the project background and the Online Indicator Data Collection Tool. The Online Indicator Data Collection Tool is a web-based survey developed by the research team that enables quick collection of food environment data using smartphones, tablets, or desktop computers. This survey is printer-friendly to accommodate participants’ data collection preferences.

#### Step 2: LEAF group chooses areas of interest

Community groups selected child-relevant settings, such as schools, childcare, recreation facilities, and other public buildings, to assess using their Mini NRC. Each setting has accompanying indicators, or “key areas where it is important to take action to improve children’s eating behaviour” ([13], p.2), which were developed under the provincial strategy [23] and occasionally adapted by the research team to fit the local context (see [16] for details).

#### Step 3: data collection

**Fig. 1:**
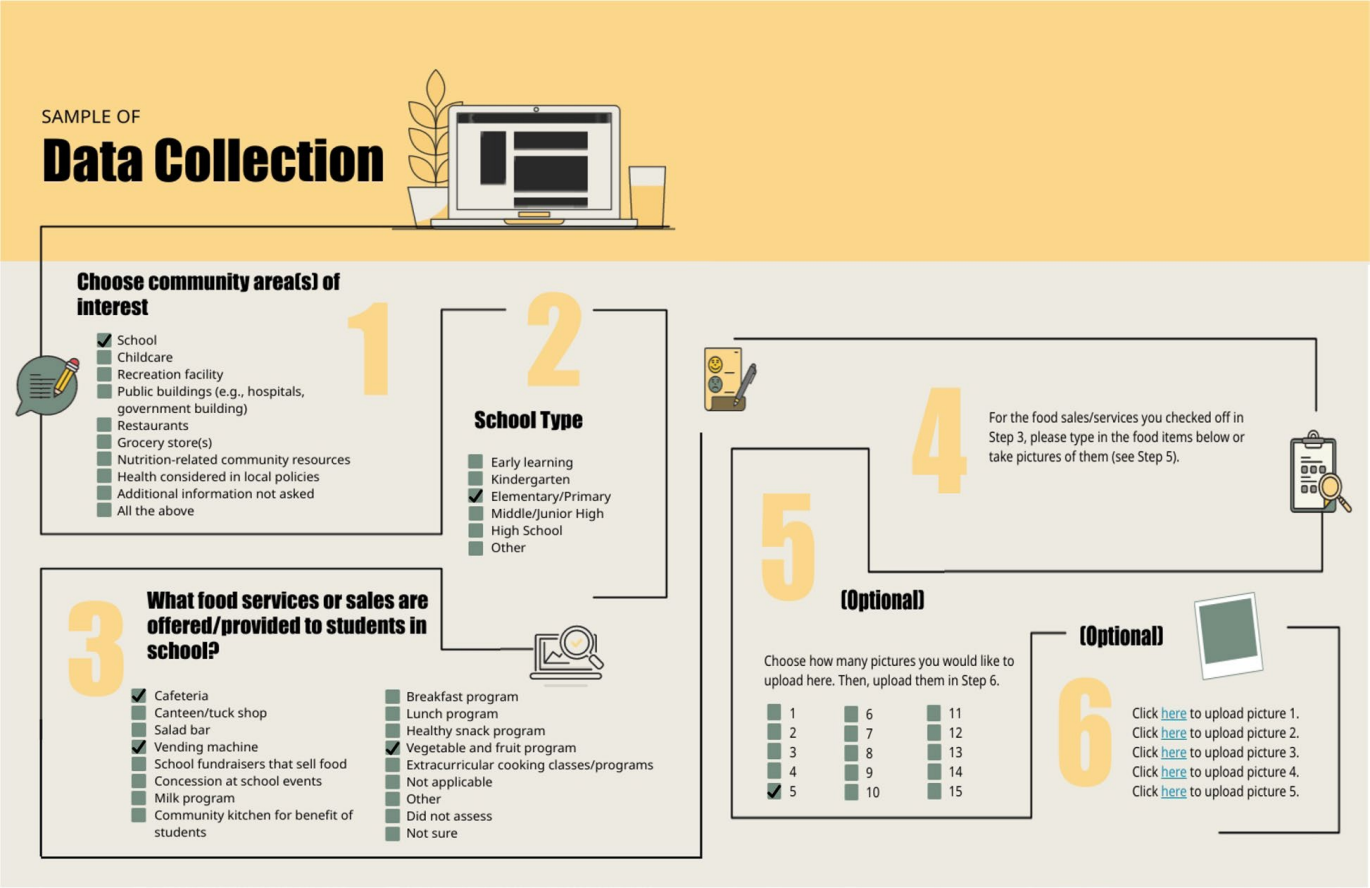
Sample of data collection for Indicator 1. Community groups enter responses for steps 1–6, providing data for Indicator 1: high availability of healthy food in school settings

Participants used the Online Indicator Data Collection Tool to collect food environment data, which included the type and cost of food available in each setting, as well as any policies, programs, and resources related to healthy eating. Community stakeholders collected food environment data by answering survey questions and uploading pictures and documents (see Fig. 1).

#### Step 4: data analysis

Once data collection was complete, the LEAF research team analyzed and graded the data (see [23] for details), creating a draft Mini NRC.

#### Step 5: validation meeting

The LEAF research team and LEAF community group met via teleconference to ensure that the draft Mini NRC represented the data collected and that no critical information was missed. The LEAF research team subsequently made necessary revisions, at times incorporating additional indicator data with further analysis and grading.

#### Step 6: recommendation meeting (with the LEAF research team)

Examining key findings and grades, the LEAF community group began developing community-specific recommendations for each indicator using the provincial NRC as a guide. This meeting was semi-structured and informal: LEAF community members provided their unique perspectives about potential actions to improve their food environments. The LEAF research team helped steer the conversation and provided input based on the provincial context.

#### Step 7: recommendation meeting (without the LEAF research team)

The LEAF community group held a follow-up meeting(s) to finalize the recommendations, which were incorporated into the community’s Mini NRC by the LEAF research team. To mitigate potential power differentials and promote open, honest conversations among LEAF community groups, the LEAF research team chose not to attend these meetings.

#### Step 8: community launch

The LEAF community group chose a strategy to share the findings from their Mini NRC, and the LEAF research team provided support. Typically, community groups hosted a public event to share and discuss Mini NRC results, recommendations, and next steps with their broader community.

## Methods

We used a constructivist qualitative collective case study design [24] to explore the process of citizen science in seven LEAF communities. Completion of the entire LEAF process was the only requirement for inclusion in this study. Thus, we enrolled communities as they completed LEAF, ending recruitment when we reached saturation in the study’s themes [25]. The resulting sample included communities that differed in many aspects (see Table 1). By including multiple communities, we hoped to gain a more in-depth understanding of how community context impacted the LEAF process. We used multiple data generating strategies, including semi-structured interviews, document review, and participant observation, to examine citizen science in the LEAF process through different lenses and subsequently gain a more holistic understanding of the phenomenon [26]. We used purposive sampling [25] to intentionally select individual participants and documents based on the amount of information they could provide about the LEAF process. Ethics approval for this study was granted from the Research Ethics Board at the University of Alberta (Pro00084508). Informed written consent was obtained from all participants prior to interviews.

### Data collection

The primary data generating strategy was individual semi-structured telephone interviews with 26 community stakeholders who were involved in the LEAF project (hereafter referred to as LEAF citizen scientists). Interviews lasted an average of 39 min and were conducted by the first author upon community completion of LEAF (between December 2018 and March 2020). The interviews addressed LEAF citizen scientists’ experiences collecting and interpreting local food environment data, as well as the process of creating community-specific recommendations for change.

We used document review and participant observation as supplementary data generating strategies. The first author completed a document review of each study community’s final Mini NRC (*n* = 7) to provide context for events that could not be directly observed [24] and to help uncover meaning relevant to the research problem [27]. In particular, community-specific recommendations provided insight into the community context and priorities. Additionally, the first author took observational field notes, guided by the research purpose and questions from LeCompete and Preissle’s [28] framework, over the same 16-month period that the interviews were conducted. Observations were recorded at LEAF meetings and at the final Mini NRC launch for five included communities. The decision to record observations was made after the first two LEAF communities had completed the LEAF process. The first author recorded her initial thoughts and interpretations, along with documenting important contextual information about the event, such as the date, time, location, length of event, and attendee’s occupation or sector of employment. Document review and participant observation helped contextualize the interview findings and guide the interview process. Regarding document review, differences between Mini NRCs, such as included settings or recommendations, highlighted questions that needed to be asked and provided prompts to further explore the LEAF process. Regarding participant observation, viewing LEAF as it unfolded enabled us to capture nuances in the process unavailable during one-on-one interviews and provided access to interesting conversations or atypical events that the first author could inquire about during interviews.

### Data analysis

We applied Charmaz’s [29] inductive constant comparison analysis strategy to all data in a multi-phase analysis process. We began by conducting a within-case analysis, where the first author employed multiple rounds of initial and focused coding, using each LEAF community as a separate case. The first author and the principal investigator met to discuss the initial and focused codes, generating preliminary conceptual categories. Next, we conducted a cross-community comparison, searching for similarities and differences between each community’s preliminary conceptual categories. The first author used Excel matrices and NVivo analytic software to facilitate cross-community comparisons and generate preliminary themes, which were discussed and finalized at meetings with the principal investigator. Throughout the entire data analysis process, the first author engaged in unstructured memo-writing to facilitate within and between community comparisons and increase data abstraction [29]. Although final themes were common across communities, we retained important community-specific details within the category headings and descriptions to reflect critical contextual differences between communities.

## Results

Analysis revealed two main themes, *process factors* and *relationship building*, each consisting of multiple interconnected categories. Process factors were influences that facilitated or hindered the LEAF process in each community. Relationship building consisted of three interconnected strategies, *engaging the right people*, *treading lightly*, and *reaching a consensus,* that communities used during the LEAF process to build and strengthen internal connections among LEAF citizen scientists, as well as external connections between LEAF citizen scientists and other community stakeholders.

### Process factors

Participants and the researcher identified several factors that influenced the LEAF process, including the local context, the flexibility of the LEAF process, and turnover among LEAF community groups. These factors will be presented here and expanded upon in the subsequent theme to demonstrate their influence on relationship building during the LEAF process.

The researcher and participants noted numerous ways that the local context influenced the LEAF process. Although all study communities shared a similar provincial political and social context, they were diverse in many ways. Communities varied in quantifiable ways, including but not limited to size, location, and average household income, and in less measurable ways, including but not limited to community priorities, culture, and history with healthy eating and food-related initiatives. Participants believed that these contextual factors shaped their experiences with LEAF and the strategies they used to create their Mini NRC. Community groups altered many aspects of the LEAF process, dictating LEAF timelines, included settings and indicators, data quantity, and Mini NRC dissemination. For example, the largest community (community E) decided not to host a validation meeting (step 5) because of the logistical concerns associated with coordinating the large group of LEAF citizen scientists.

Although participants generally viewed LEAF’s flexible approach as a strength, enabling them to engage more community members in the process (discussed below), they also described several resulting limitations. For example, some participants expressed concerns regarding the lack of data requirements and long project timelines, which had implications for perceived data accuracy and relevancy. As explained by one participant,

I think the challenge I saw with the process was, because it was over a period of time, by the time it came to analyzing the data, some of it was already outdated. You know, for example, current situations that were in schools when the data had been collected, then through government changes, funding changes, grant changes, it wasn’t necessarily reflective of what was currently happening, which was a challenge (P17).

Participants described turnover within LEAF community groups as a barrier that disrupted report continuity and, at times, led to confusion regarding the purpose or process of LEAF. For example, one participant stated that, “when this project first started, I didn’t quite know what was going on. So, I didn’t know what I was collecting the data for in the beginning” (P4). The extent and impact of turnover varied among communities, which was in part due to the differing approaches to community engagement. For example, the impact of turnover on report continuity was felt most acutely in a community that did not have a pre-existing community group leading LEAF. In comparison, one community that utilized a pre-existing community group cited continuity as a facilitator for the LEAF process:

We consistently would meet, or we would have teleconferences… And so, we just called it BFE, so Benchmarking Food Environments. [BFE] was always a standing agenda item, so we could say where we were at, who was doing what (P2).

### Relationship building

Analysis revealed that relationship building was a vital but challenging component of the LEAF process. Participants used three interconnected relationship-building strategies, represented by the categories *engaging the right people, treading lightly,* and *reaching a consensus,* to strengthen community relationships and build capacity to improve food environments that impact children and youth.

#### Engaging the right people

Participants reported strategically engaging other community stakeholders, such as those with authority to influence policies and programs in settings that impact children and youth, to strengthen the LEAF process and build community relationships. Many participants believed that widespread community engagement enabled them to create feasible recommendations and was an indicator of the community’s commitment to food environment action. As explained by one participant, “So, I think where we’ve seen that more diverse data collection, I think you’re ultimately going to get more buy-in. ‘Cause it shows more buy-in right from the get-go—if everybody’s willing to put their time in and it’s not just the dietitian time doing it [data collection]” (P14). The researcher and some participants also noted the positive implications that widespread community engagement could have for relationship building. For example, LEAF meetings provided a venue for networking and building community partnerships. As explained by one participant,

The people that were in the room really helped… like sort of spark some of those recommendations because… people were there from those organizations. So, it kind of makes you think like, ‘oh yeah, why couldn’t we partner with them to talk a little bit more about this [creating healthy food environments for children and youth]?’ (P15).

In each community, engagement in the LEAF process was diverse and multi-sectoral. For example, communities had representation from schools, recreation facilities, childcare centres, the government, public health, etc. Participants described several recruitment tactics they used to increase engagement of hard-to-reach groups, such as decision-makers and policy influencers. Recruitment strategies were particularly salient in Community E, where LEAF project leads described drawing on their strong, pre-existing community connections by sending recruitment emails through community coalitions’ listservs and engaging decision-makers in separate one-on-one consultations. LEAF’s flexible approach also seemed to facilitate recruitment: flexible timelines permitted the time-consuming process of engaging community members; flexible data requirements and methods (e.g., submitting pictures as food environment data) reduced participants’ workload and enabled individuals without extensive nutrition knowledge to collect data.

Participants and the researcher noted how the level of community engagement affected the strategies that communities used during the LEAF process. One illustrative example was communities’ perceived need to tread lightly, a sentiment more strongly expressed by communities that lacked representation from the settings assessed in their Mini NRC.

#### Treading lightly

Participants described proceeding carefully during the LEAF process, attempting to minimize possible misinterpretations of their Mini NRC and prevent defensiveness from other community stakeholders. Reasons for treading lightly varied among participants, settings, and communities, and included a community or setting’s history with healthy eating initiatives; perceptions of nutrition as a sensitive topic, involving community culture and values; local, demographic factors (such as community size or rurality); and the level of community engagement in LEAF. To illustrate the overlapping and additive nature of these factors, we will briefly compare Community C, where the need to tread lightly was pervasive, with Community G, where the need to tread lightly was minimal.

Participants described Community C as having a contentious history with healthy eating initiatives. For example, they reported that some setting stakeholders have resisted making nutrition-related changes despite an existing agreement to do so. Although the reasons for this resistance were complicated and not fully understood by participants from Community C, one participant cited historical tensions between stakeholders involved in creating this agreement as a contributing factor. Additionally, Community C was unable to retain strong representation from settings assessed by their Mini NRC, such as schools, for various reasons including competing school priorities that vied for stakeholders’ time. These factors, along with the local culture, led to a strong perceived need to tread lightly:

I don’t know if it’s this town specifically or just rural in general, but there is just such a culture around, ‘we’re going to the rink [ice hockey arena], and we’re having our rink food.’^1^ And that can be a barrier because, again, people get a little defensive about like, ‘well, that’s our way of life.’ And, in going through this process—you know, there’s a lot of beef farmers in the area—and I know some people were personally offended by the fact that the guidelines don’t promote the overconsumption of red meat. So, you know, treading carefully and not making people feel uncomfortable or that their way of life is threatened. I think it’s really important out here for maintaining those relationships (P5).

In contrast, participants from Community G expressed less need to tread lightly, especially with issues concerning schools. Here, community culture and stakeholder engagement in LEAF seemed to play an essential role. For example, one participant described Community G’s government as having a “forward thinking” approach to health and wellness initiatives. The influence of stakeholder engagement on the LEAF process was validated by a decision-maker from Community G, who described how their participation fostered self-reflection, which included reflecting on whether their school’s current practices supported their goal of promoting healthy eating environments for students. For this participant, self-reflection and their overall engagement in the LEAF process helped to minimize their defensiveness and increase ownership over the changes that needed to be made:

To be part of the process is, I think, really important. Because… I think if someone had come in and done it [creating the Mini NRC] … I think then you become a little more—resentful is not the right word—but it’s like, you know, being told by someone else outside, ‘Oh okay, yes. One more thing you expect us to do at schools... Whereas, when we were part of the process right from the beginning, I think it was good for us to… self-reflect. And look at what [we’re] doing in [our] own practices.’ (P21)

Participants and the researcher noted three strategies that communities employed to tread lightly: using sensitive language, respecting what exists, and obtaining permission. First, communities used language as a tool, consciously attempting to use inoffensive wording and highlight the need for community partnerships. For example, Mini NRC recommendations often used words like “support,” “encourage,” “work with,” and “engage.” Second, communities attempted to recognize, include, and build on community strengths. For example, two sections of the Mini NRC (“on the horizon” and “local assets” sections) were designed to highlight upcoming local changes and existing community programs or supportive policies respectively. Lastly, communities sought out permission and feedback from relevant stakeholders during the LEAF process. Although permission from included settings was not always necessary (e.g., collecting data from publicly accessible settings), some participants equated consent for data collection with commitment to food environment action. As stated by one LEAF project lead, “well, if you don’t get consent, then I don’t think they’re [the setting] interested in participating and making changes. So, then what’s the point [of collecting data in these settings]?” (P16). The local context seemed to influence communities’ desire to obtain consent from included settings, with community size playing an influential role. In general, there was less potential for anonymity in smaller communities, since the community may only have one or two schools, restaurants, or recreation settings. In some smaller communities, this lack of anonymity influenced the desire to gain consent. As described by one participant,

There are very few restaurants in that community. So, just wanting them to understand how the data would be used, so that they didn’t feel that there’d be comparisons among different establishments, or that the data would be used in a way that could be perceived sort of negatively on them (P20).

#### Reaching a consensus

Participants perceived LEAF as an informal process, consisting of discussion and consensus reaching. Reaching a consensus involved integrating two types of knowledge: local knowledge, which was broad and community-specific, spanning factual information about available resources to understandings of community values, perceptions, and upcoming changes; and outside knowledge, which referred to contextual information about the province’s food environments and knowledge derived from other communities (both LEAF and non-LEAF communities). Within each community, the consensus-reaching process was affected by the level of turnover among LEAF citizen scientists. For example, when a new stakeholder joined, LEAF groups had to rehash previous discussions and decisions. As explained by one LEAF project lead,

A person would attend, and then they wouldn’t attend two meetings, and they show up to the fourth meeting, and it’s like, ‘Oh well I don’t want to do this.’ Like, ‘Why did you guys do this?’ And we’ve all discussed it at the first two meetings, and they weren’t involved (P16).

Participants and the researcher felt that local knowledge influenced the settings, indicators, and recommendations that were included in a community’s Mini NRC. For example, although municipalities have the authority to implement healthy zoning policies, such as restricting unhealthy food retailers within 500 m of schools, at least one community rejected this recommendation due to local knowledge:

One of the examples that always came up is, ‘[Community C]’s really long and skinny. And everything is located in one area.’ So the schools are all located next to each other and they’re all located [close] to the downtown. So, in terms of like whether unhealthy food is within so many meters [of the school] … right now, it’s not something that they can do much about (P5).

Although participants often stressed that recommendations needed to be feasible, they had difficulty articulating *how* they determined what was feasible. They did, however, note the importance of local knowledge:

We just, I guess, know that [what is practical and relevant] about our community... And just knowledge of the community in itself—who’s there and in what positions. I guess its all very community specific. So, if you have the wrong person working in the recreation centre, it won’t work, right. (P25)

The influence of outside knowledge varied widely across included communities. Although all included communities had access to provincial-level knowledge via the LEAF research team, they had different opportunities for integrating learnings from other communities. For example, communities that participated in LEAF at the start of the research project could not review and learn from previous LEAF communities’ completed Mini NRCs. Further, LEAF communities seemed more likely to communicate with other communities within their administrative health zone. Within our sample, this suggests that communities in Central Alberta had more opportunity to learn from other LEAF communities, a notion that was supported by participant interviews. In fact, LEAF project leads in Central Alberta described connecting with one another during monthly meetings:

The dietitians will share where they’re at in the process, and they’ll share things like what’s working well… or what successes they’ve had in the community, as far as changes they’ve been able to implement... So, the benefit of that is just the ability [for others] to say, ‘Oh! Okay, I didn’t think of that;’ ‘Oh, that might be an opportunity for us as well.’

## Discussion

The LEAF project supported the creation and application of food environment evidence: it enabled residents to collect and interpret local food environment data, develop realistic recommendations for change, and provided them with an evidence-based advocacy tool to support the implementation of these recommendations. Analysis of the LEAF process revealed two main themes, process factors and relationship building, which provide important implementation insights.

Relationships were an important aspect of the LEAF project in two ways: positive community relationships facilitated and enhanced the LEAF process, and community engagement in the LEAF process promoted further relationship building. Participants considered relationship building a vital but challenging component of the LEAF process, a finding consistent with results from related citizen science intervention studies [30–32]. Indeed, upon reflection, we recognized that pre-existing relationships between LEAF project leads and the LEAF research team through prior community-based research projects formed a strong foundation for initiating the project and helped build momentum for other communities’ participation. Pre-existing, trusting relationships between LEAF project leads and local residents helped some communities achieve widespread engagement across a diverse range of sectors. Although prior relationships were present in all communities, those with weaker connections, such as Community C, often dedicated more time and effort to ensure their Mini NRC did not offend community stakeholders. We found that widespread community participation facilitated the research process by reducing individual workload and enhanced research outputs by generating contextually appropriate Mini NRCs that contained feasible recommendations. Locally generated knowledge may be particularly relevant for complex public health issues, such as food environments, where there is no gold standard intervention for all contexts [6, 33, 34]. Integrating the knowledge, perspectives, and values of local residents could mitigate community resistance to change and provide new pathways for creating healthy food environments for children and youth. For example, due to intimate knowledge of their community design, Community C recognized the impracticality of implementing zoning bylaws, which was a provincial recommendation, and sought out alternative actions to achieve the same goal. Additionally, citizen science interventions could create new solutions for complex health problems by spurring the development of new partnerships or strengthening existing partnerships [9, 13]. For example, the LEAF project brought stakeholders from different sectors together to discuss the challenges and possibilities for improving their food environments. At times, these conversations prompted LEAF citizen scientists to form new partnerships amongst themselves to reduce the barriers preventing change. For example, at a LEAF meeting in one community, conversations between a childcare association and the local foodbank led the group to recommend that the foodbank should provide childcare centers with fresh produce; this recommendation would provide mutual benefits, enabling the childcare centers to increase the amount of fruits and vegetables available to children, while also reducing the foodbank’s current food waste issues.

Our study contributes to the extant literature by identifying three interconnected strategies, engaging the right people, treading lightly, and reaching a consensus, that communities used to build relationships while employing a citizen science food environment research approach. These strategies provide insight into the complexities and challenges of building community relationships while conducting citizen science research, which are best demonstrated through a closer examination of the category treading lightly. Based on our experiences with the provincial NRC [18], we expected that communities might have some concerns with assigning grades to community settings. However, we did not anticipate that these concerns would lead communities to alter the LEAF process. For example, to avoid offending community stakeholders, LEAF communities often obtained permission to collect data, used sensitive language to stress the need for community partnerships, and attempted to build on existing community strengths. Collectively, these strategies suggest that a strengths-based, capacity building approach to citizen science food environment research could prove beneficial. To apply this approach, citizen scientists would use the LEAF process to identify and enhance work that community stakeholders are already doing to improve children’s eating environments. A strengths-based approach is consistent with best practices of community-based obesity prevention [35] and aligns with previous work in citizen science food environment research [31].

The LEAF process and relationship building were shaped by numerous process factors, including the local context, the flexibility of the LEAF process, and turnover among LEAF community groups. Each community’s local context, including their values and priorities, demographic factors, and history with healthy eating initiatives, influenced the LEAF process. Because of this, participants often perceived LEAF flexibility as a strength, enabling them to tailor the approach to their local context. For example, participants appreciated the multiple data collection methods and flexible timelines and data requirements, which enabled them to increase community engagement in the citizen science process. However, some participants outlined limitations to this flexibility, including concerns regarding outdated or incomplete data. The LEAF process was contingent upon community stakeholders’ ability to meet, which at times meant months-long delays due to scheduling conflicts or competing priorities within the community or LEAF research team. The issue of incomplete data demonstrates the importance of having the ‘right’ stakeholders involved: that is, individuals with intimate knowledge of the setting that can collect accurate and complete data. Although we used Validation Meetings (step 5) to increase data accuracy, any additional data that was collected, analyzed, and graded as a result of this meeting further prolonged the whole LEAF process. Concerns about data accuracy and reliability are common in citizen science [31, 36] and could have important implications for the impact of citizen science interventions. In our study, for example, if community stakeholders perceived the data in their Mini NRC as flawed, they might reject the results and recommendations for food environment action. Thus, although often a strength, the possible limitations of intervention flexibility (i.e., incomplete or inaccurate data) should be taken into consideration when developing and implementing citizen science community-based interventions.

### Limitations

This study has several limitations. First, results primarily represent the perspectives of individuals from the first seven LEAF communities that agreed to participate in this study. Included communities and participants often supported healthy eating initiatives; therefore, they may have viewed the LEAF process more positively than other individuals or communities. Another limitation of this research is the small sample size within each community, which limited our ability to examine the contextual factors that influenced citizen science during the LEAF process. We had a limited number of potential participants to draw from because turnover among LEAF citizen scientists created recruitment difficulties. We attempted to mitigate this limitation by including at least one LEAF project lead from each community because they could provide detailed information about all aspects of the LEAF process. Lastly, although our sample included a range of diverse communities, the transferability of our study’s findings may be limited by the shared provincial political and social context. For example, communities’ perceived need to tread lightly may have been heightened due to the prominence of beef cattle farming in Alberta, given that dietary guidelines suggest limiting red meat consumption.

## Conclusion

Participants’ experiences using the citizen science approach revealed the importance of relationships and the intricacies of these relationships as crucial to the LEAF process. We suggest that individuals leading citizen science approaches take time to consider strategies that could aid in building vital relationships and strive to strengthen these connections throughout the research process. Results also support the need for sustainable and adaptable intervention approaches that can be modified to fit the local context. We recommend a web application that enables residents to complete food environment assessments without the support of university researchers, which could help to build the necessary evidence base for policy and program development. Finding ways to stimulate and sustain local food environment action is essential, given that diet, which is influenced by food environments, continues to be a leading behavioural risk factor for chronic disease.

## Data Availability

The datasets generated and analyzed during this study are not publicly available to preserve participants’ confidentiality but are available from the corresponding author upon reasonable request.

## Abbreviations

BFE: Benchmarking food environments
CBPR: Community-based participatory research
LEAF: Local environment action on food
Mini NRC: Mini nutrition report card on food environments for children and youth
Provincial NRC: Alberta nutrition report card on food environments for children and youth

## Footnotes

1 In this context, the term “rink food” refers to fast food items, such as fries and hot dogs, that are nutrient-poor and energy-dense.
